# Measuring spatial and temporal properties of visual crowding using continuous psychophysics

**DOI:** 10.1167/jov.25.7.7

**Published:** 2025-06-13

**Authors:** Dilce Tanriverdi, Frans W. Cornelissen

**Affiliations:** 1 Laboratory for Experimental Ophthalmology, University Medical Center Groningen, University of Groningen, Groningen, Netherlands

**Keywords:** continuous psychophysics, visual crowding, spatiotemporal dynamics, peripheral vision

## Abstract

Visual crowding refers to the difficulty in recognizing objects in the periphery when surrounded by clutter. Traditional trial-based paradigms, while effective in measuring spatial aspects of crowding, do not capture the temporal dynamics involved. In this study, we assessed the feasibility of a continuous psychophysics paradigm that measures both the spatial extent and temporal processes of visual crowding. Eight participants continuously tracked the orientation of a rotating Landolt C while the distance between the target and a ring-shaped flanker varied systematically over time. Participants set a reference stimulus to match the orientation of the target. The paradigm included “jump-points,” where the orientation of the target suddenly shifted, allowing us to measure the recovery rate of participants’ tracking errors following these disruptions. Tracking accuracy was compared between flanked and isolated conditions. Additionally, participants’ report errors were used to assess both the crowding extent and the temporal recovery rate from the jumps, with the crowding extent results compared with those obtained from a conventional trial-based version of the paradigm. The recovery rate was calculated by fitting an exponential decay function to participants’ report errors after the jumps. The results showed that the crowding extent measured using the continuous paradigm was consistent with that obtained using trial-based methods and aligned with Bouma's rule. Moreover, flankers decreased both tracking accuracy and recovery rate following the jumps. These results demonstrate that our continuous psychophysics paradigm is useful for measuring the spatiotemporal aspects of crowding.

## Introduction

Visual crowding refers to the phenomenon where surrounding clutter or flanking stimuli make it more challenging to perceive a target object, particularly in peripheral vision, where the visual system struggles to isolate and process individual elements within a crowded scene (Bouma, 1970). Understanding and measuring visual crowding are crucial for gaining insights into underlying neural mechanisms and have practical applications in areas such as reading, driving, and interface design (Dobres, Wolfe, Chahine, & Reimer, 2018; Whitney & Levi, 2011). Moreover, visual crowding effects are reported to be more pronounced in people with certain visual or developmental deficits such as amblyopia (Bonneh, Sagi, & Polat, 2007; Greenwood et al., 2012; Levi, 2008; Levi, Hariharan, & Klein, 2002), nystagmus (Chung & Bedell, 1995; Tailor, Theodorou, Dahlmann-Noor, Dekker, & Greenwood, 2021), dyslexia (Geiger & Lettvin, 1987; Martelli, Di Filippo, Spinelli, & Zoccolotti, 2009; Pel, Boer, & van der Steen, 2019; Spinelli, De Luca, Judica, & Zoccolotti, 2002), and glaucoma (Ogata et al., 2019; Shamsi, Liu, & Kwon, 2022; Stringham et al., 2020).

Traditional methods for studying crowding typically involve trial-based paradigms, where participants are required to identify a target that is surrounded by one or more flankers. These methods, while precise, are often time consuming (Pelli, Palomares, & Majaj, 2004; Pelli et al., 2016; Tanriverdi & Cornelissen, 2024). Measurements across various target–flanker eccentricities require a series of trials to accurately determine the crowding extent; therefore, studying crowding in developmental, clinical, and other non-conventional populations is an important challenge to clinicians and researchers alike (Levi, 2008; Pelli et al., 2016; Verghese, McKee, & Levi, 2019).

Continuous psychophysics, a relatively recent development, offers an alternative approach to traditional trial-based methods. Continuous target-tracking psychophysics simultaneously provides information about the time course of visual processing (Bonnen, Burge, Yates, Pillow, & Cormack, 2015). With this technique, a feature of a stimulus (e.g., its orientation) is tracked via an input device. Changes in another stimulus property of interest (e.g., in case of crowding, this could be target–flanker distance) can then be associated with changes in the tracking performance. How this performance changes with the magnitude of the stimulus property of interest allows one to make inferences about various aspects of visual processing.

Previous research has shown that, by using continuous psychophysics, one can make near-identical estimates of visual processing metrics compared with traditional trial-based methods while additionally gaining an understanding of the temporal processes related to the visual function in question (Ambrosi, Burr, & Cicchini, 2022; Ambrosi, Burr, & Morrone, 2024; Ambrosi, Pomè, & Burr, 2021; Bhat, Cicchini, & Burr, 2018; Bonnen et al., 2015; Bonnen, Huk, & Cormack, 2017; Burge & Cormack, 2024; Grillini, Ombelet, Soans, & Cornelissen, 2018; Grillini, Renken, & Cornelissen, 2019; Huk, Bonnen, & He, 2018; Soans et al., 2021; Straub & Rothkopf, 2022; Vrijling et al., 2023).

Previously, we developed a semicontinuous psychophysical paradigm where participants had to make continuous eye movements to static crowded targets (Tanriverdi & Cornelissen, 2024). This paradigm proved to be faster and as reliable as traditional forced-choice paradigms for healthy and young participants but proved to be challenging for both older and glaucomatous participants (Tanriverdi, Al-Nosairy, Hoffmann, & Cornelissen, 2024). Motter and Simoni (2007) analyzed gaze tracking records in monkeys during continuous search to infer crowding distance, demonstrating another way in which continuous measurements can provide insights into crowding effects. However, to our knowledge, a continuous psychophysics paradigm where stimulus properties change over time has not been implemented to measure visual crowding.

The main goal of this study was to demonstrate the feasibility of using continuous psychophysics to measure visual crowding. Our paradigm was inspired by the work of Harrison and Bex (2015), Harrison and Bex (2017), where participants were asked to report the orientation of a Landolt C stimulus using a reference stimulus while the Landolt C was flanked by a ring-shaped stimulus. In our novel approach, instead of having discrete trials, participants continuously reported the orientation of a rotating Landolt C while the target–flanker distance varied continuously over time. Using this paradigm, we measured the crowding extent and investigated the test–retest reliability of this novel approach. We also compared the crowding extent values obtained from the continuous crowding paradigm to those derived from a conventional trial-based version of the paradigm. Moreover, as mentioned earlier, one of the key advantages of continuous psychophysics is its ability to track visual phenomena over time, providing insights into the temporal processes underlying these phenomena. To leverage this, we introduced “jump points” into the continuous movement of the target orientation, where the target gap suddenly shifted to a different orientation. These jump points allowed us to observe how quickly participants recovered from this interruption in the continuous motion. We hypothesized that the presence of flankers would slow down this recovery process compared with the isolated condition. In other words, in addition to the decreased accuracy, we expected participants to take longer to return to the pre-jump level of report accuracy in the flanked condition compared with the isolated condition.

## Methods

### Participants

Eight participants took part in the experiment (five females, three males). The age range of the participants was 25 to 61 years, with a mean age of 33 years. All had normal or corrected to normal vision (self-reported). If corrected to normal, participants used their own glasses or contact lenses during the experiment. All participants other than the two authors (DT and FWC) were naïve to the purpose of the experiment. The study received approval from the ethical committee of the University Medical Center Groningen and adhered to the tenets of the Declaration of Helsinki.

### Equipment

The experiments were executed using MATLAB software (MathWorks, Natick, MA) on a MacBook Pro (mid-2015; Apple, Cupertino, CA) running macOS Monterey 12.3.1. Stimuli were presented on an LED backlight monitor (ZOWIE XL2540; BenQ, Taipai, Taiwan) with a refresh rate of 144 Hz and a pixel resolution of 1920 × 1080. The mean luminance of the screen was 63 cd/m². The screen dimensions were 52.5 cm × 29 cm, yielding a pixel density of approximately 36.57 pixels/cm horizontally and 37.24 pixels/cm vertically. Visual stimuli were created using functions coded in Psychtoolbox for MATLAB (Brainard, 1997; Kleiner et al., 2007; Pelli, 1997). Eye position signals were recorded with an EyeLink 1000 (SR Research, Ottawa, ON, Canada) at a sampling rate of 1000 Hz. Calibration of the eye tracker was performed using the EyeLink built-in nine-point calibration procedure, and the eye tracker was controlled and integrated into the experimental script using the EyeLink Toolbox (Cornelissen, Peters, & Palmer, 2002). One eye of the participant was recorded, selected based on which eye the EyeLink system accepted most easily during the calibration process. Participants viewed the display binocularly from a distance of 64 cm, with a fixed head position using a head and chin rest.

### Stimulus and paradigms

**Figure 1.**
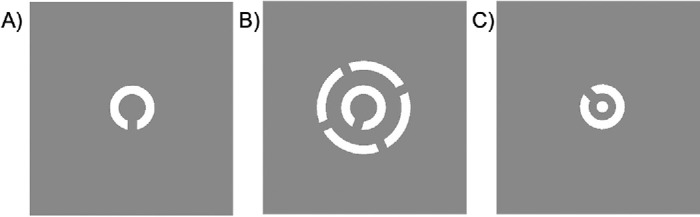
Stimuli presented to participants during the experiment. (**A**) The isolated target. (**B**) The flanked target. (**C**) The reference stimulus.

The design of the stimulus was inspired by the paradigm described by Harrison and Bex (2015), Harrison and Bex (2017). For both trial-based and continuous paradigms, the stimuli consisted of a white Landolt C target placed at the 10° eccentricity to the left of the fixation point on a gray background. The target was 2° in diameter with a 0.4° line and gap width. The target either was presented in isolation (Figure 1A) or was surrounded by a ring stimulus with four openings with a 0.4° line and gap width, as can be seen in Figure 1B. The distance between the outer edge of the target from the inner edge of the flanker ring ranged from 0° to 5°. The fixation was a white dot with a 0.5° diameter placed in the middle of the screen (Figure 1C).

#### Selection of target and flanker gap orientations

For both the trial-based and continuous paradigm, the gap orientation of the target was randomly drawn from a uniform 360° distribution for each presentation. Similar to Smithers, Shao, Altham, and Bex (2023), the flanker gap orientations were determined as follows: One orientation was randomly selected from a normal distribution centered around the target gap orientation with a 45° standard deviation. The other three gaps were placed at 90° intervals relative to this selected orientation. Participants used a reference Landolt C, identical to the target, placed in the middle of the screen to report the perceived orientation of the target (Figure 1C). Participants moved the mouse to the right to rotate the reference clockwise and to the left to rotate the reference anti-clockwise.

#### Continuous paradigm

In the continuous crowding paradigm, the above indicated orientations of the stimuli were chosen as the starting points. During a trial, the stimulus rotated continuously either clockwise or counterclockwise. The rotation speed of the target and flanker was 72°/s (0.5° per screen refresh rate). If a flanker was present, it rotated at the same velocity as the target. In the flanked conditions, the flanker was initially presented at a target–flanker distance of either 0° or 5°. This distance then either increased or decreased continuously with a speed of 0.13°/s. When the target–flanker distance reached certain preselected values (0.31°, 0.35°, 1.35°, 2.20°, and 3.37°), the orientations of the target and flanker gaps were reassigned randomly according to the above procedure. This reassignment caused a noticeable jump in the otherwise continuous movement of all the gaps, requiring participants to re-identify the gap orientation of the target. These jumps were also presented at the same time points for the isolated conditions. After each jump, the direction of the stimulus rotation (clockwise or counterclockwise) was randomly reselected to prevent prediction and keep participants alert. The preselected target–flanker distances were originally chosen to be equally spaced on a logarithmic scale between 0.8° and 5.8°, where 0.8° represents the inner-border-to-outer-border separation when the target and flanker were touching. To express distances as center-to-center values, we redefined 0.8° as 0° and subtracted it from all distances.

During the trials, participants were instructed to keep their gaze fixed on the fixation spot and to adjust the orientation of a reference stimulus to match the orientation of the target by continuously moving the mouse. The gaze of the participants was monitored using the eye tracker. If participants moved their eyes more than 1° away from the center of the fixation point, the trial was paused, and the stimulus was removed from the screen. The fixation dot turned red to signal this fixation break until fixation was re-established. This fixation check served to keep the eccentricity of the target stable. Blinks were handled differently to avoid unnecessary disruptions. When a blink occurred, the program retained the last recorded eye position rather than pausing the trial, as pausing for each blink would have been impractical during the 40-second trial durations. This approach allowed participants to blink naturally without compromising the experimental procedure.

In one block, the flanked condition was repeated eight times. In four of these, the target–flanker distance gradually increased, and in the other half it gradually decreased. The increasing and decreasing conditions were meant to balance each other. The isolated condition was repeated four times. Because the timing of the jumps varied depending on whether the invisible target–flanker distance was increasing or decreasing, in half of the isolated trials the jumps were presented as the invisible flanker was decreasing and in the other half as if it was increasing. One trial lasted for 40 seconds. After each trial, participants were instructed to press a button to start a new trial. This was done to provide ample opportunity to rest in between trials. All conditions were presented in random order.

#### Trial-based paradigm

In the trial-based paradigm, the gap orientations were static. The procedure for selecting gap orientations was the same as the continuous paradigm. The target was presented either in isolation or with a flanker placed at one of the seven distances (0°, 0.31°, 0.35°, 1.35°, 2.20°, 3.37°, or 5°). The stimulus and the reference appeared on the screen at the same time, similar to in the continuous paradigm. Participants were instructed to keep their gaze at the fixation spot. The eye movement procedure, including the handling of blinks, was identical to that in the continuous paradigm: If participants moved their eyes more than 1° away from the fixation spot, the trial was paused, the stimulus was removed, and the fixation dot turned red to signal a fixation break. The trial resumed when fixation was re-established, ensuring that the eccentricity of the target remained stable. For blinks, the program retained the last recorded eye position to avoid unnecessary interruptions, allowing participants to blink naturally without compromising the experimental procedure. The participants were instructed to adjust the orientation of the reference Landolt C to match the perceived orientation of the target by moving the mouse and clicking the left button to submit their responses. After participants responded, the stimulus and the reference disappeared, and a new trial began. Each stimulus condition was repeated 25 times, totaling 200 trials.

### General procedure

The study was conducted at the University Medical Center Groningen in a dimly lit room. Two paradigms (continuous and trial-based) were completed in different blocks. Each participant completed the trial-based paradigm once and the continuous tracking paradigm twice. The order of the trial-based and continuous paradigms was randomized for each participant. Before starting each paradigm, the experiments were explained to the participants, and they completed a demo session to ensure that they understood the relevant task. This demo session presented the stimulus only in the isolated condition and ended when participants confirmed that they understood and were able to perform the task. Each block began with calibration of the eye tracker.

### Data analysis

The main dependent variable for both the continuous and the trial-based paradigms was the report error, defined as the difference between the orientation of the stimulus and the participant's response, ranging from –180° to 180°.

#### Continuous paradigm

For the continuous paradigm, we calculated the absolute value of the report errors, providing a measure of the magnitude of participants’ errors irrespective of the direction of this deviation. The report errors collected from the eight flanked conditions, with half of the conditions flipped to maintain consistent decreasing and increasing flanker response rates, were averaged together, as well as those from the four isolated conditions.

Before the analysis, the raw and report error data were cleaned to remove “artifactual errors” caused by the jumps in target orientation and participants’ inability to control the mouse perfectly. This process involved detecting peaks in the response errors specifically within the isolated condition in the report error data. Peaks were identified using a threshold method. The threshold to identify a peak was set at twice the standard deviation of the response errors in the isolated condition, ensuring that only dominant peaks above the typical variability were detected. When they had been identified, the corresponding data segments—both in the isolated and flanked conditions—were marked for deletion if the response error exceeded the mean error + 1 *SD* in the isolated condition. The data were deleted from both conditions to maintain consistency in the availability of the data. On average, this excluded about 10% of the data in a given trial.

To assess the similarity between the presented orientations and the participants’ responses, we computed the cosine similarity for both the isolated and flanked conditions. We used the cosine similarity as a standardized way to analyze participants’ ability to track the target. Unlike absolute report error, which can vary widely depending on the scale of the data, cosine similarity is a scale-invariant measure that directly reflects the accuracy of the participant's tracking in relation to the orientation of the target. Cosine similarity between the stimulus orientation and the response orientation was calculated using the following equation:

CosineSimilarity=∑i-1n[cos(θstimulus,i)·cos(θresponse,i)∑+sin(θstimulus,i)·sin(θresponse,i)]∑i-1n[cos2(θstimulus,i)·sin2(θstimulus,i)·∑i-1n[cos2(θresponse,i)·sin2(θresponse,i)

where θ*_stimulus_*_,_*_i_* and θ*_response_*_,_*_i_* represent the orientations of the stimulus and the participant's response, respectively, in radians. Note that we use the magnitude of the vectors in the denominator due to the circular nature of the data. This metric provides a measure of how closely the participant's response matched the stimulus orientation, with values closer to 1 (the maximum value) indicating higher similarity. The cosine similarity is also referred to as tracking performance throughout the manuscript. We compared the tracking performance of the isolated and flanked conditions to determine whether the introduction of flankers impacted the participant's ability to track the orientation of the target.

To quantify the crowding extent, we performed a linear regression analysis on the report error data. Separate linear regression models were fitted to the report error data from both the isolated and flanked conditions. The slope and intercept of these regression lines were used to determine their point of intersection, representing the crowding extent—the critical distance at which the presence of flankers begins to impair the perception of the target stimulus.

To determine the time it took participants to return to a stable level of report error following a jump, we fitted decay functions to the report errors following the jumps. Specifically, we selected the first 2080 ms of report errors after each jump (300 report errors after each jump) and averaged these errors for each run of the continuous paradigm for each participant in both the flanked and isolated conditions. An exponential decay function was then fitted to the averaged report errors:

$$\begin{array}{c} E\left(t\right)=ae^{-bt}+c \end{array}$$

where *a* reflects the typical initial magnitude of the error across all jumps, *b* indicates how quickly the error typically stabilizes across all jumps, and *c* represents the baseline level to which the error stabilizes. We used the parameter *b* to determine the recovery rate of each participant for each condition. We referred to this variable as the recovery rate throughout the manuscript. A higher recovery rate would represent a faster recovery rate from the jump.

It is known that the average motor response time to a stimulus ranges between 200 and 500 ms (Woods, Wyma, Yund, Herron, & Reed, 2015). To observe whether the motor response time affected the estimated recovery rates, we removed the first 40 report errors (∼280 ms), thus keeping the 40th to 300th errors after the jump and again fitted the exponential decay function to these data. This resulted in an estimate of the recovery rate that took an average motor latency period into account.

#### Trial-based paradigm

To quantify crowding in each stimulus condition, we used the report errors to calculate the circular standard deviation for each target–flanker distance and isolated conditions. This involved employing maximum-likelihood estimation to fit a Von Mises circular distributions to the observers’ report errors within each condition, using the *mle.vonmises* function from the *circular* package (version 0.5-0) (Agostinelli & Lund, 2023) in RStudio (R Foundation for Statistical Computing, Vienna, Austria). This measure is referred to as the “perceptual error,” similar to how this has been used in previous literature (Harrison & Bex, 2015; Harrison & Bex, 2017; Smithers et al., 2023).

Using the perceptual errors, we performed a regression analysis by fitting a regression line to the perceptual errors in the flanked condition. We then conducted a hinged line analysis to calculate the point on the *x*-axis at which this line intersected the baseline perceptual error line, thereby determining the crowding extent for each participant.

### Statistical analysis

First, tracking performance for the isolated and flanked conditions was compared for the first and second runs of the continuous paradigm. A 2×2 repeated-measures ANOVA with within-subject variables—Flank Condition (Flanked vs. Isolated) and Trial Run (First vs. Second)—was conducted with tracking performance as the dependent variable.

Additionally, crowding extent values for the continuous paradigm and the trial-based paradigm were compared. First, the crowding extents from the first and second runs of the continuous paradigm were compared using a paired-samples *t*-test. As they did not show a significant difference (see Results section), the mean of these values was calculated. Subsequently, a paired-sample *t*-test was conducted to compare the crowding extent between the trial-based and continuous paradigms. We then examined whether there was a correlation between the trial-based paradigm and the continuous paradigm concerning crowding extent. A Pearson correlation analysis was performed among the crowding extent of the trial-based paradigm, the mean crowding extent of the continuous paradigm, and the crowding extent from the first and second runs of the continuous paradigm.

We conducted a 2×2 repeated measures ANOVA with within-subject variables—Flank Condition (Flanked vs. Isolated) and Trial Run (First vs. Second)—on the recovery rate, and on the recovery rate while taking motor latency period into account. Moreover, to determine if there was a difference in recovery time between isolated and flanked conditions, *t*-tests were conducted on the average report error data following the jump points across trials for each participant and condition. These tests compared the mean report errors over the last 50 data points (which we took to represent the level of stable performance) to the earlier error values following the jump. Starting from the first data point after the jump, the first point where the *t*-test indicated that the difference was non-significant was identified as the time point of recovery.

## Results

**Figure 2.**
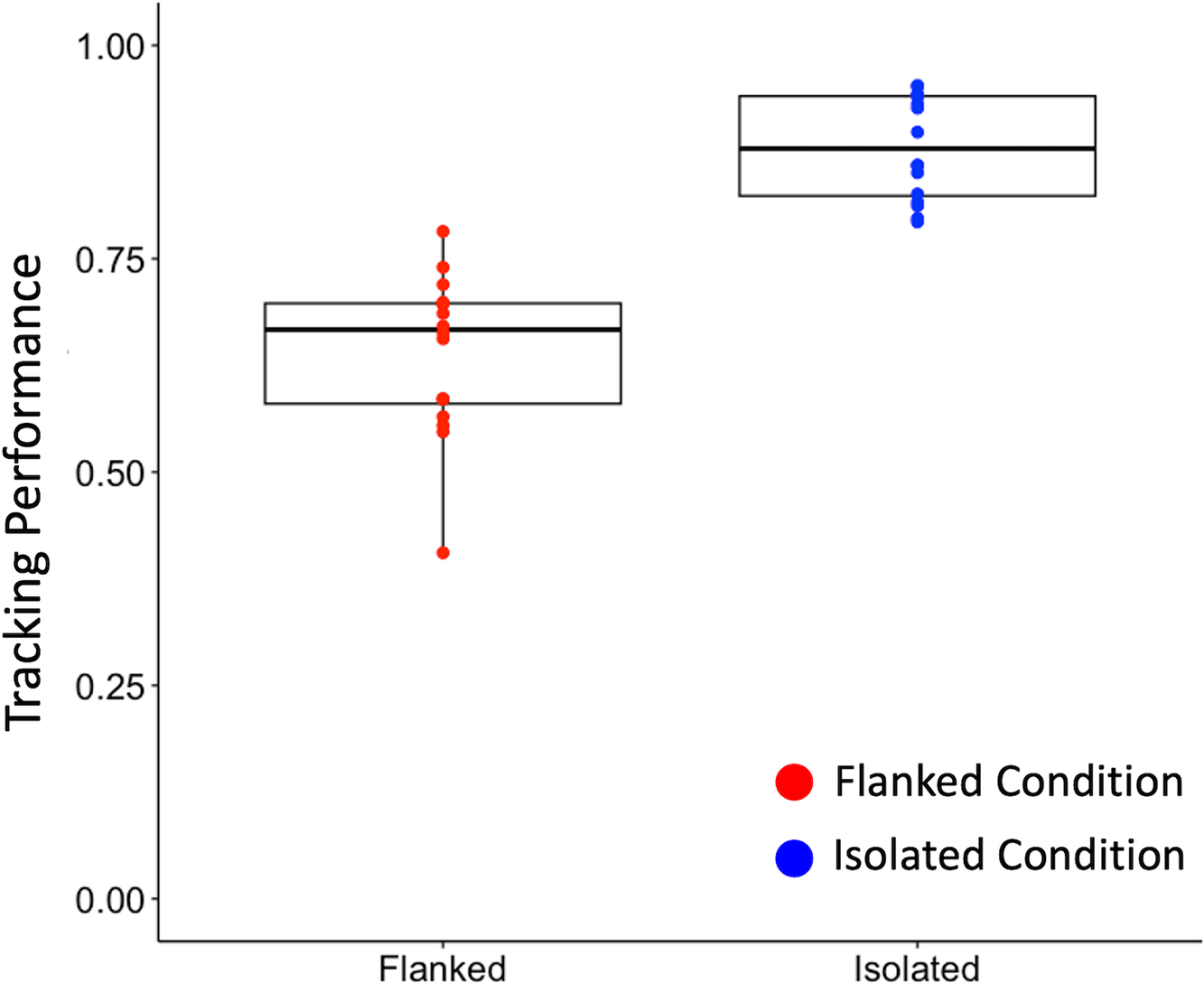
Tracking performance (cosine similarity) between the target and response orientations for the flanked (red) and the isolated (blue) conditions. The dots on the box plots represent the individual observers’ data points, and the thick line and boxes indicate the median and interquartile range (IQR), respectively. Note that, for the flanked condition, all target–flanker distances were combined to calculate the tracking performance.

We started our analysis by comparing the tracking performance for the isolated and flanked conditions in the continuous paradigm. As shown in Figure 2A, participants were better able to follow the target orientation when the target was isolated (*M* = 0.88, *SD* = 0.061) compared with when it was flanked (*M* = 0.64, *SD* = 0.09). The 2×2 repeated ANOVA results revealed that there was a significant effect of the flankers, *F*(1, 7) = 107.73, *p* < 0.001, $\eta_{p}^{2}$ = 0.94. There was no effect of trial run, *F*(1, 7) = 0.97, *p* = 0.36, $\eta_{p}^{2}$ = 0.12, nor an interaction, *F*(1, 7) = 0.3, *p* = 0.6, $\eta_{p}^{2}$ = 0.04.

**Figure 3.**
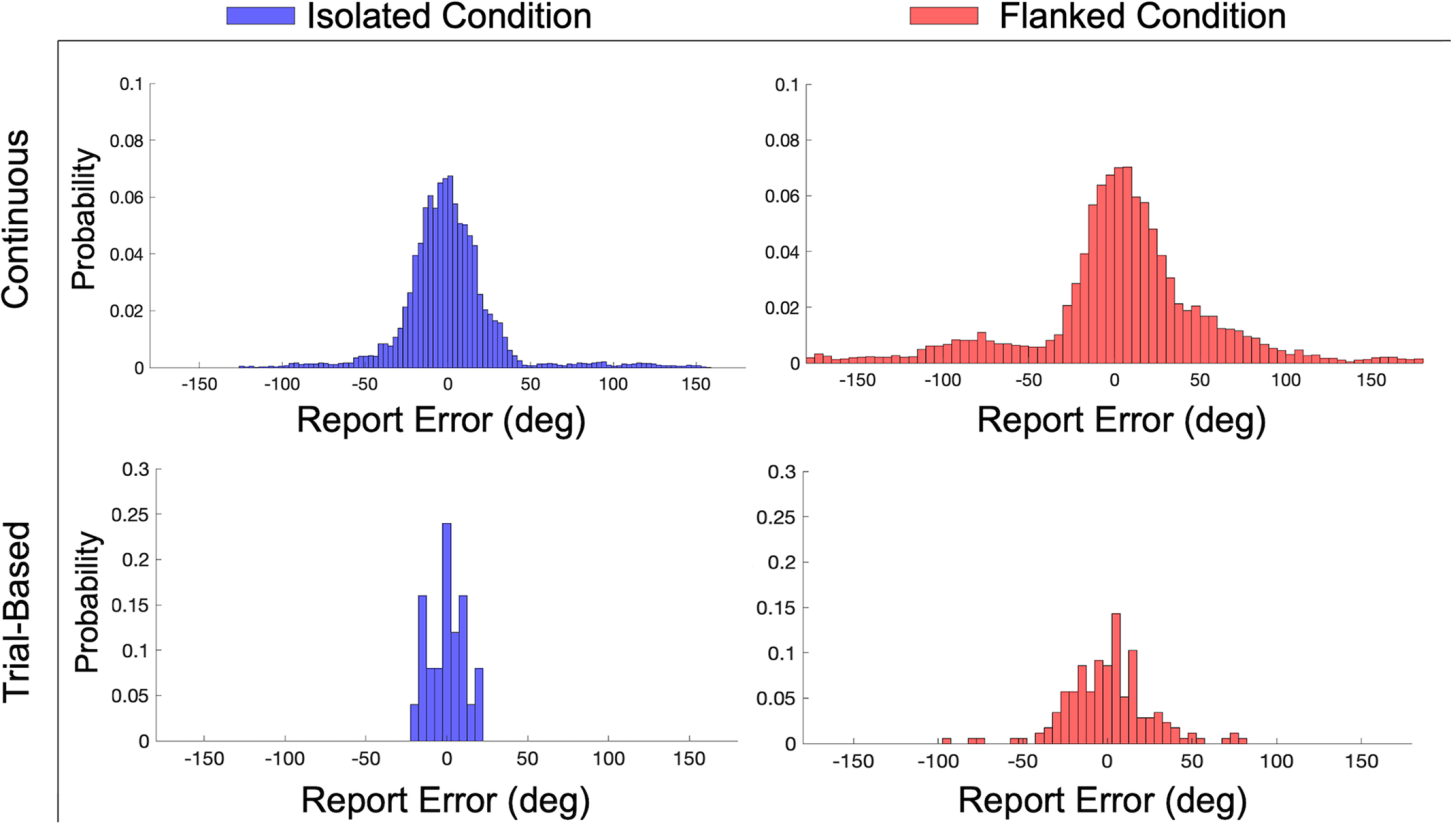
Histograms showing the relative distributions of the report errors for the isolated (blue) and flanked (red) conditions in the continuous paradigm and the trial-based paradigms. Note that the histograms show the distribution of report errors before taking their absolute value for further analyses. Moreover, the report errors of all flanked conditions are grouped together.

**Figure 4.**
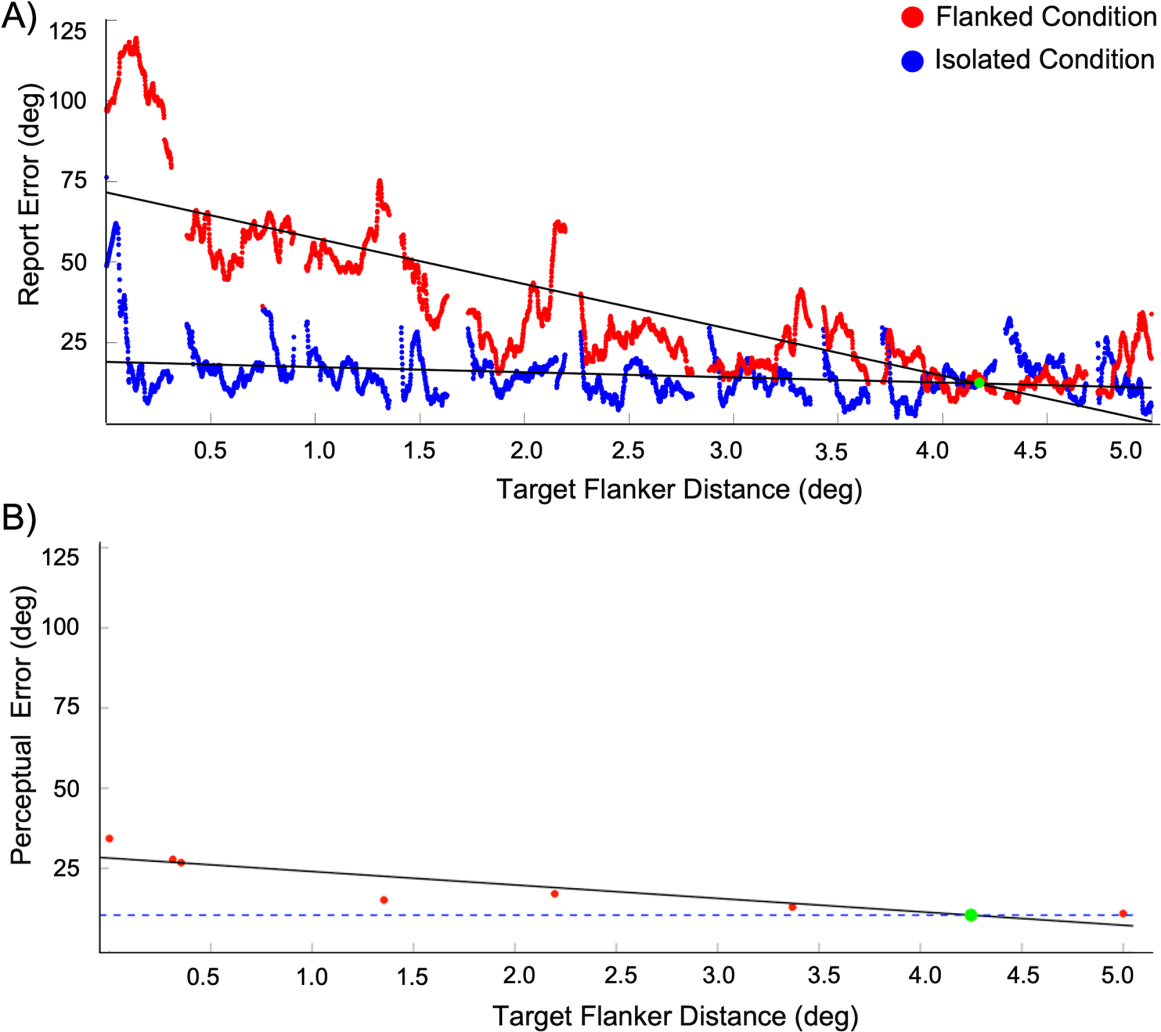
(**A**, **B**) The crowding extent analysis for the continuous paradigm (**A**) and the trial-based paradigm (**B**). Panel A displays the absolute report error as a function of target–flanker distance in the continuous paradigm. Separate linear regression lines were fitted to the flanked (red) and isolated (blue) data, with their intersections (marked by a green dot) indicating the estimated crowding extent. Panel B displays the perceptual error measured in the trial-based paradigm. Here, the perceptual error, as estimated in separate conditions, is plotted as a function of target–flanker distance. A regression line was fitted to the flanked (red dots) data. The intersection of this line with the error level in the isolated condition (blue dashed line), again marked by a green dot, represents the crowding extent derived from the trial-based data. The data shown are example data from a single participant (DT).

Next, we calculated crowding extent based on the report errors. To demonstrate this process, we first plotted the report errors (Figure 3) of a single participant (DT) in both the isolated (blue) and the flanked (red) conditions in the continuous (Figure 3A) and trial-based (Figure 3B) paradigms. In both paradigms, it can be observed that the response error distributions were broader in the flanked conditions. Moreover, in Figure 4, we illustrate how linear regression was used to estimate crowding extent in both the continuous and trial-based paradigms.

**Figure 5.**
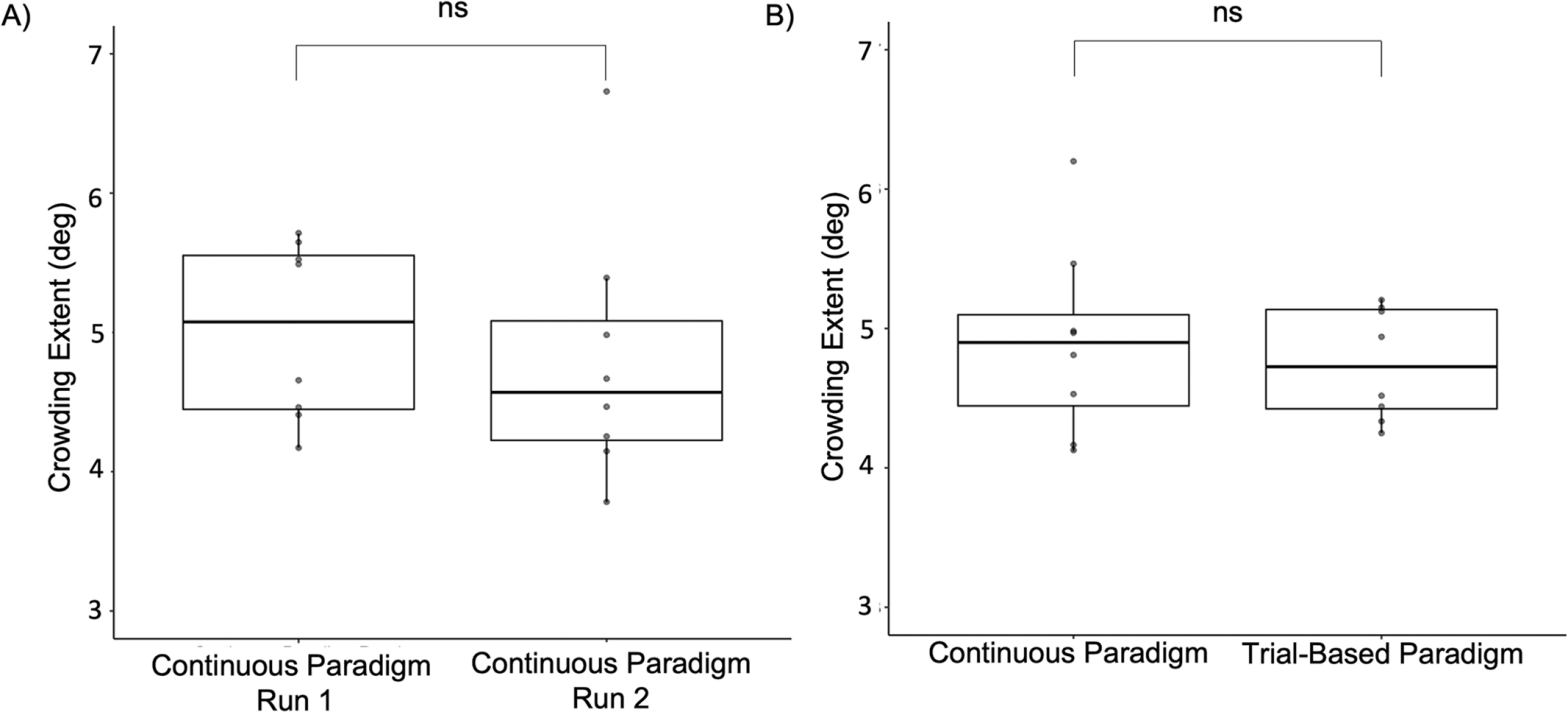
Crowding extent in continuous and trial-based paradigms. (**A**) Crowding extent calculated from the first and the second run of the continuous paradigm. (**B**) Crowding extent from the trial-based paradigm and the mean crowding extent from the continuous paradigm. The dots on the box plots represent the individual data points, and the thick lines and boxes show the median and IQR, respectively.

**Figure 6.**
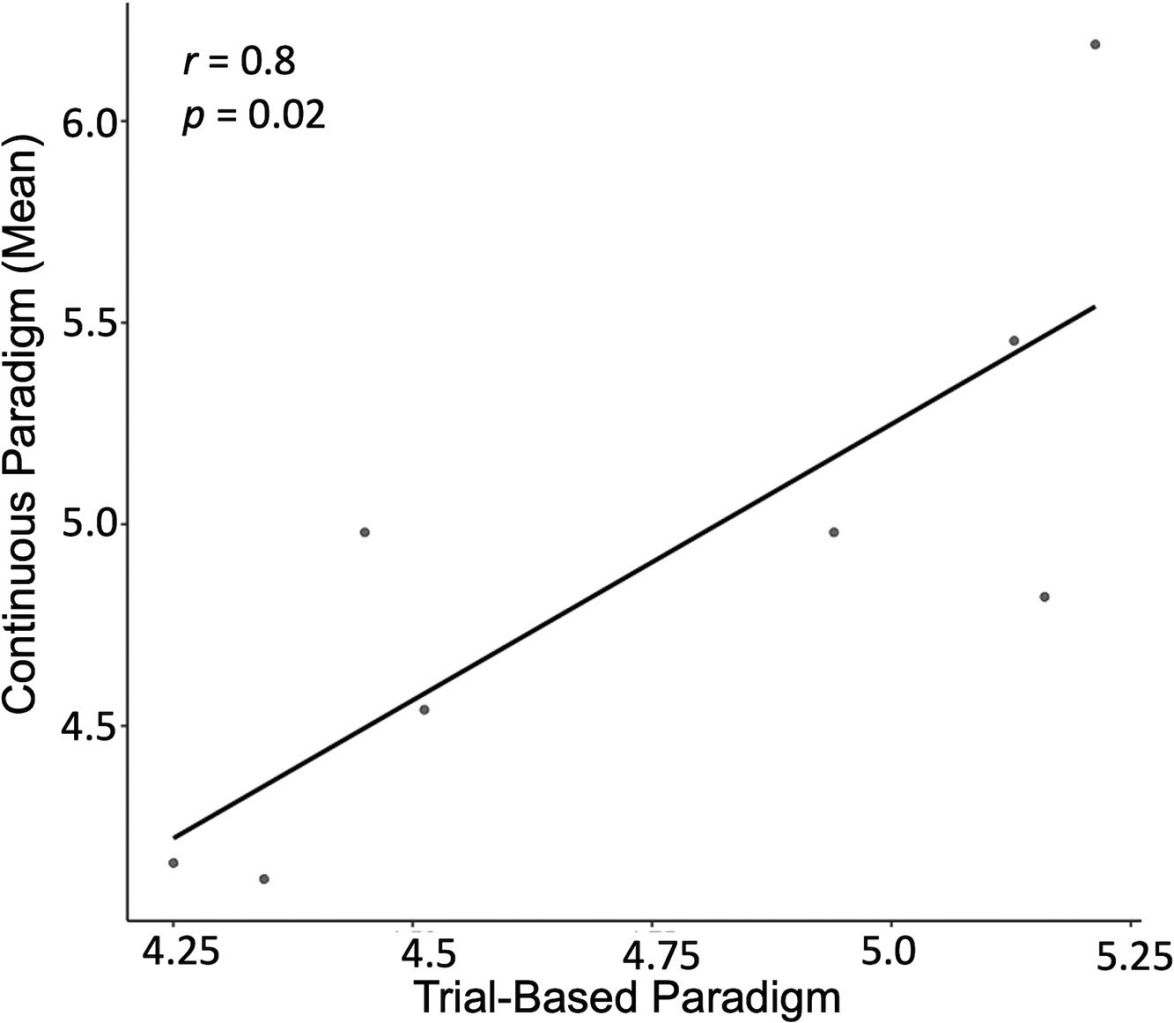
Correlation of crowding extent between the continuous and trial-based paradigms. Each point represents an individual participant. For the continuous paradigm, crowding extent was calculated based on two runs, each lasting approximately 10 minutes. The trial-based paradigm consisted of a single run lasting approximately 10 minutes.

To compare the estimated crowding extents between the paradigms, first we examined whether the crowding extent differed between the two runs of the continuous paradigm. As shown in Figure 5A, crowding extent did not differ significantly between the first and second run of the continuous paradigm, *t*(7) = 0.72, *p* = 0.5, *d* = 0.25. Therefore, when comparing the estimated crowding extents between the paradigms, we took the mean crowding extent of the two continuous runs. As can be seen in Figure 5B, the mean estimated crowding extents in the continuous and the trial-based paradigms did not differ significantly, *t*(7) = –1.01, *p* = 0.35, *d* = –0.36. Figure 6 shows that, when we compared the estimated extents in both paradigms at the individual participant level, we found a significant correlation between the crowding extent in the continuous and trial-based based paradigms (*r* = 0.8, *p* = 0.02).

Additionally, we analyzed the spatial extent of crowding separately for the conditions in which the flankers approached the target and moved away from the target. A paired *t*-test revealed no significant difference between the estimated crowding extents in the shrinking and receding conditions, *t*(7) = 1.45, *p* = 0.19, *d* = 0.51.

**Figure 7.**
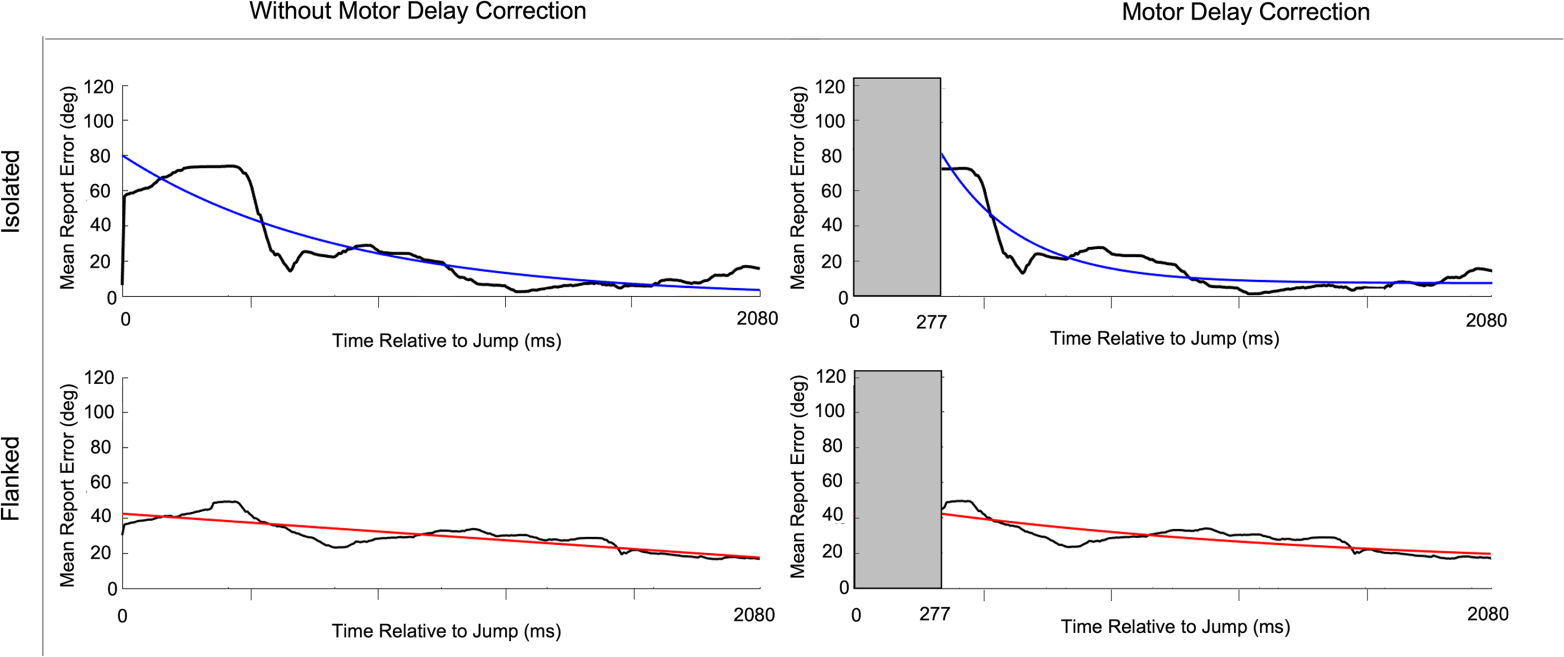
Recovery toward baseline report error following a sudden change in the target orientation (a jump). In all panels, the black line represents the observed report error over time, averaged over all jumps, with the colored curves representing the exponential decay function. Data are for a single observer (DT). In the “without motor delay correction” column, we included the entire 2080-ms time window following the jump when fitting the decay function. In the “motor delay correction” column, we omitted the initial 40 data points (∼280 ms) to mitigate a (generic) likely influence of a motor response latency.

One of the potential advantages of using continuous psychophysics is that it allows estimating temporal aspects of the studied phenomenon. Here, we fitted an exponential decay function to the average report error following a sudden change in the orientation of the target (the “jump”). Based on these decay functions, we can derive the recovery rate. A higher recovery rate would mean a faster rate of return to the base level report error following the jump. To illustrate the process of estimating the recovery rate, Figure 7 shows the report error data over time, as well as the associated decay functions of a single participant. As can be seen in Figure 7, the report error following the jump appeared lower in the flanked condition. This was a general pattern observed for all participants, despite assigning random magnitudes of jumps and having similar average jump sizes between conditions. This difference can be attributed to the higher baseline error in the flanked condition. Because the flankers already impaired the participants’ ability to track the target, the additional error due to a jump was less noticeable. In contrast, in the isolated condition, where tracking was generally more accurate, jumps caused more noticeable errors, but the participants’ recovery rates were quicker.

**Figure 8.**
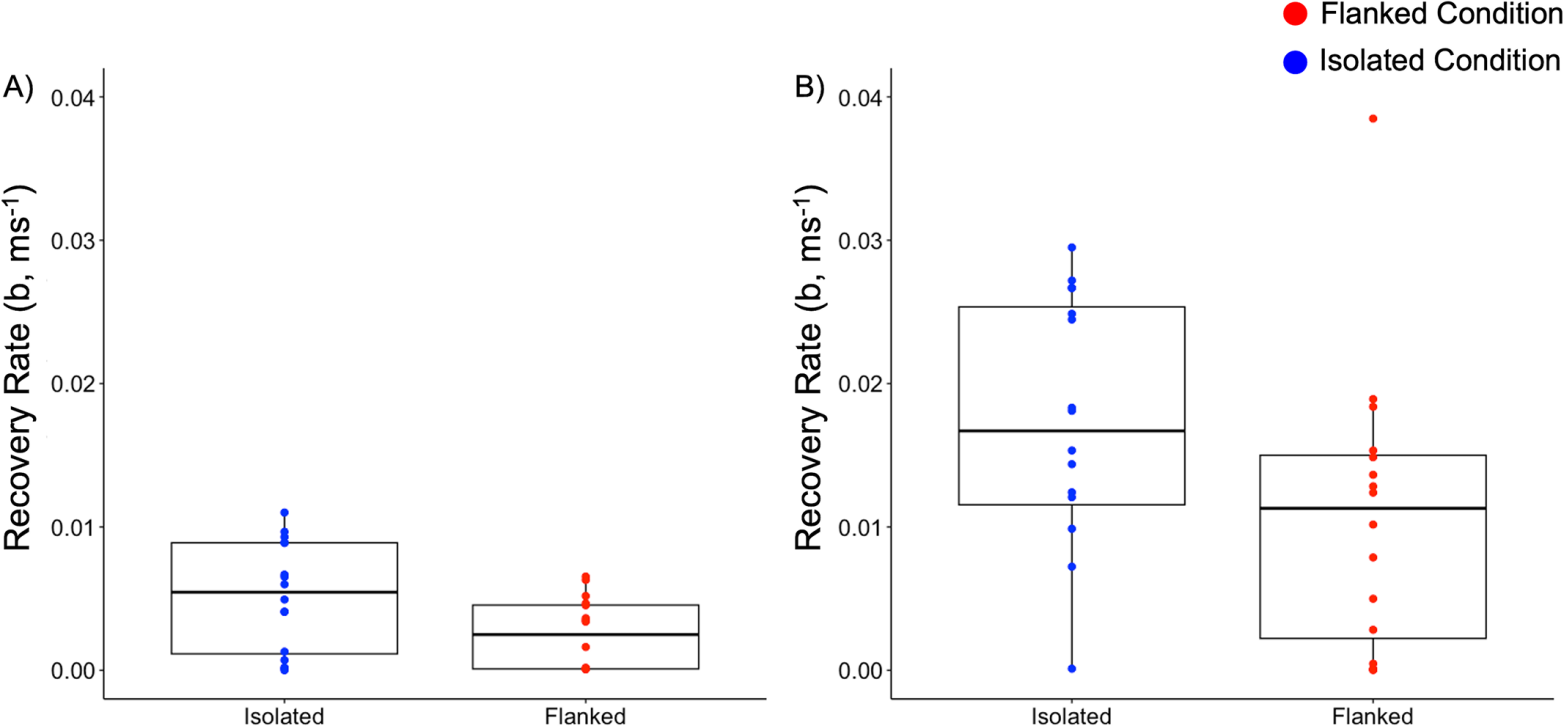
Recovery rate in the continuous paradigm. (**A**) Rates in flanked (red) and isolated (blue) conditions, not taking into account the motor delay period. (**B**) Recovery rates in the flanked and isolated conditions taking into account a generic ∼280-ms motor delay. The dots on the box plots represent the individual data points, and the thick lines and boxes show the median and IQR, respectively.

Figure 8 shows the recovery rates in the continuous paradigm, for the isolated and flanked conditions and taking into account whether or not a motor delay is considered. Our results indicate that, in both situations, participants recovered faster in the isolated compared with the flanked conditions—without: *F*(1, 7) = 8.27, *p* = 0.02, $\eta_{p}^{2}$ = 0.54; with: *F*(1, 7) = 14.46, *p* = 0.01, $\eta_{p}^{2}$ = 0.67.

**Figure 9.**
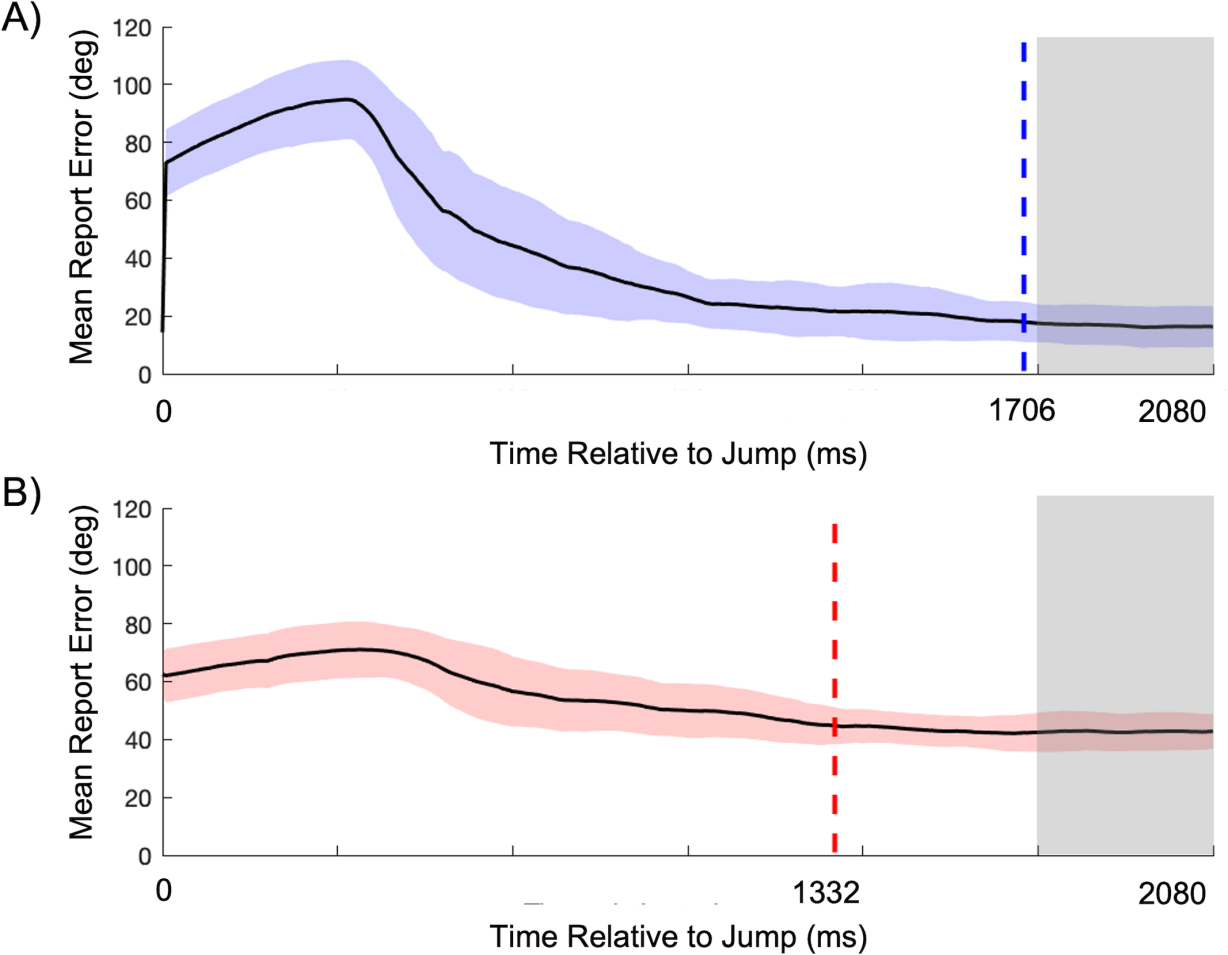
Recovery time analysis for the continuous paradigm in isolated (blue) and flanked (red) conditions. (**A**) Data for the isolated condition. (**B**) Data for the flanked condition. In both panels, the black line represents the observed report error after the jump point, averaged across all jumps and participants. The shaded areas around the data points indicate ±1 *SD*. The dashed line marks the recovery point. The gray rectangle represents the section we took as reference for the stable error rate.

Figure 9 shows the results of the recovery time analysis. The analyses indicated that, in the isolated condition, the report error returned to its stable level 1706 ms after the jump, on average. In the flanked condition, this was around 1332 ms after the jump. Thus, on average, in the presence of flankers, the report error returned to its stable level 374 ms earlier than when the target was presented in isolation. Note that the initial errors in the isolated condition were higher than in the flanked condition. This is discussed in more detail in the Discussion section, where we consider baseline error levels and recovery dynamics across conditions.

## Discussion

In this study, we showed that it is feasible to study the dynamics of visual crowding using a novel continuous psychophysics paradigm. Our key findings are that (a) target orientation tracking performance was reduced when the target was flanked compared with when it was presented in isolation, indicating that crowding affected participants’ performance; (b) the continuous paradigm effectively measures crowding extent, yielding results comparable to those obtained through a conventional trial-based method; and (c) in the flanked condition, tracking errors exhibited a slower temporal decay, stabilizing at a higher baseline level earlier, whereas, in the isolated condition, errors showed a larger initial rebound and took longer to reach the lower baseline level. Our results highlight the utility of a continuous paradigm in studying both the spatial and temporal aspects of the visual phenomenon of crowding.

### Flankers decrease tracking performance

The results from our continuous crowding paradigm showed that the mean tracking performance was 88% in the isolated condition and 64% when the flanker was present, measured by the cosine similarity. This indicates that participants were able to maintain a more accurate alignment with the orientation of the target when it was not surrounded by distracting flankers. This finding aligns with existing literature on visual crowding, where surrounding elements typically interfere with the perception of a central target, particularly in peripheral vision, likely due to the integration or confusion of target and flanker features in the visual processing system (Bouma, 1970; Greenwood, Bex, & Dakin, 2010; Harrison & Bex, 2017; Levi, 2008; Whitney & Levi, 2011).

### Crowding extents measured with continuous and trial-based paradigms are comparable

Our study demonstrated that our continuous crowding paradigm can be used to measure the extent of crowding, yielding results that are comparable to those obtained through a conventional trial-based paradigm. We showed that the mean crowding extent measured with the continuous paradigm (5) was not different from the mean crowding extent measured with the trial-based paradigm (4.75). Moreover, we showed that these measures were correlated with each other on the individual participant level (*r* = 0.8). These results support the feasibility of using a continuous psychophysics paradigm to measure crowding.

The crowding extent measured using the continuous paradigm not only was similar to the trial-based paradigm we employed in this study but also was consistent with the previous literature (Bouma, 1970; Kurzawski et al., 2023; Pelli et al., 2004). Our findings on crowding extent align well with Bouma's rule, which posits that the critical spacing, or the minimum distance at which crowding effects occur, is approximately 0.5 times the eccentricity of the target from the point of fixation (Bouma, 1970). In our study, the target was at 10° eccentricity, and we found that the crowding extent—where the presence of flankers began to significantly impair target perception—was 5°. This agreement between our findings and Bouma's rule provides further validation that our continuous paradigm is a reliable tool for measuring crowding extent.

### Faster stabilization but lower recovery rate in the flanked condition compared with the isolated condition

Following the sudden changes in target orientation, the analysis of post-jump errors revealed an interesting pattern: Whereas the rate of stabilization—reflected in the slope of the error reduction—was higher in the isolated condition, tracking performance returned more quickly to baseline levels in the flanked condition (around 1332 ms after the jump, compared with 1706 ms in the isolated condition). In our analysis, we noted that various aspects may underlie these somewhat counterintuitive and seemingly conflicting findings. The first aspect we noted is that the average error right after the jump was lower in the flanked condition compared with the isolated condition, despite similar average jump sizes between both conditions. This discrepancy can be explained by the higher baseline error in the flanked condition. As participants already were struggling to accurately track the target due to the presence of flankers, the additional error introduced by a jump was relatively smaller, thus reducing the impact of the jump. In contrast, when tracking in the isolated condition—where participants generally performed better—the jumps introduced more noticeable errors. Yet, in the isolated condition, the participants recovered at a higher rate from these larger errors. Thus, participants in the isolated condition could more efficiently return to their pre-jump performance levels because their overall tracking was more accurate.

Existing literature suggests that, when the visual system is given more time to process crowded stimuli, this appears to mitigate some of the crowding effects, potentially through mechanisms such as spatial and temporal integration (Chung, 2016; Lev & Polat, 2015; Tripathy & Cavanagh, 2002) and attention (Grubb et al., 2013; Kewan-Khalayly, Migó, & Yashar, 2022; Kewan-Khalayly & Yashar, 2022). The slower rate of error reduction in the presence of flankers aligns with the notion of participants attempting to integrate over a longer time period to optimize their performance. However, integrating continuously changing stimulus information over a longer period of time also risks degrading performance. This highlights the dynamic nature of visual processing, where the benefits of prolonged exposure can be counteracted by changes, which may particularly affect crowded conditions.

Moreover, studies have shown that the spatial extent of crowding increases with brief stimulus presentations (Tripathy, Cavanagh, & Bedell, 2013), a finding that may relate to the slower rate of error reduction observed in our flanked condition. This could suggest that the participants required additional time to process the influence of the flankers, consistent with the notion of extended spatial integration under dynamic conditions. Additionally, “temporal crowding,” where successive stimuli presented in close temporal proximity interfere with target perception, has been observed particularly in peripheral vision (Bonneh et al., 2007; Tkacz-Domb & Yeshurun, 2021; Yeshurun, Rashal, & Tkacz-Domb, 2015). In our paradigm, the introduction of jump points—where the target orientation shifts suddenly—may induce a form of temporal crowding. This, in turn, might explain the higher error rates in the continuous paradigm compared with the trial-based paradigm, as the participants had to contend with both spatial crowding from flankers and temporal interference from the continuously changing target orientation.

### Limitations and future research

Although our study demonstrated the feasibility of the continuous psychophysics paradigm in assessing visual crowding, there are various limitations and possible modifications that should be considered. First, the use of a mouse as a tracking device might have somewhat hindered the tracking performance of some of the participants. Participants had to move the mouse right or left, which was not directly corresponding to the circular movement of the target and reference stimuli. The use of different input devices, such as a rotating steering wheel or a joystick, could potentially provide more intuitive control and enhance tracking accuracy. Future research can compare the effectiveness of different tracking devices. The standardized cosine similarity measure provides a quantification that facilitates this comparison.

Additionally, our present study applied a fixed rotation speed of the Landolt C, a fixed trial duration, and specific timings for the jumps. Our study goal was to show the feasibility of using a continuous psychophysics paradigm for measuring crowding. In all likelihood, the continuous psychophysics paradigm can be optimized for efficiency. For example, in our current paradigm, we collected 5760 data points per 40-second-long trial. During this trial, only five jump points were introduced, but we now believe these may be the most informative moments, notably for studying the temporal aspects of crowding. Future paradigms could be made more effective by decreasing the trial time or increasing the number of jumps per trial. However, it is important to note that increasing the frequency of jumps might alter the crowding effect, as preliminary observations suggest that presenting only jumps could potentially disrupt crowding. This disruption may occur because rapid orientation changes, or transients, attract attention to the target location (Posner, Synder, & Davidson, 1980), facilitating the segregation of the target from its flankers and thereby diminishing the crowding effect (Yeshurun & Rashal, 2010). Therefore, the optimal frequency of the jumps presented requires further investigation. The velocity of the target orientation change and the frequency of the jump could be a way to study what is optimal for integrating, given the specific temporal stimulus characteristics. Related to this, we explored whether crowding extent differed between approaching (shrinking) and receding (expanding) flankers, as theories suggest that motion-induced positional shifts (De Valois & De Valois, 1991; Dakin, Greenwood, Carlson, & Bex, 2011; Maus, Fischer, & Whitney, 2011) or visual system lag effects (Williams, Phillips, & Sekuler, 1986) could influence crowding due to the direction of flanker motion. However, we did not find a difference between motion directions. Future research could explore whether adjusting parameters in the continuous paradigm (e.g., flanker velocity) enhances the ability to investigate motion-induced positional shifts or lag effects in crowding. Further examination of these effects would also strengthen the potential of the continuous paradigm as a tool for studying dynamic crowding, which more closely reflects real-world visual environments.

If the tracking can be made more intuitive and the assessment time required for reliably estimating crowding extent can be optimized, a continuous crowding paradigm may be suitable for collecting large amounts of high-quality data from virtually any observer. This would be particularly advantageous for work with developmental, clinical, and other non-traditional populations (Grillini, Renken, Vrijling, Heutink, & Cornelissen, 2020; Soans et al., 2021; Vrijling et al., 2023).

Moreover, visual crowding is known to have various distinguishing properties, such as target–flanker similarity (Andriessen & Bouma, 1976; Chung, Levi, & Legge, 2001; Levi, Toet, Tripathy, & Kooi, 1994; Levi et al., 2002; Nazir, 1992), radial–tangential difference in extent (Feng, Jiang, & He, 2007; Petrov & Meleshkevich, 2011; Toet & Levi, 1992), and asymmetries in the visual field (He, Cavanagh, & Intriligator, 1996). Future research could investigate whether such well-known properties can be replicated using a continuous psychophysics paradigm. For measuring some of these aspects, a stimulus with individual flankers (such as the Landolt C, gratings, or letters) could be beneficial. Moreover, the continuous psychophysics paradigm provides a good framework for investigating whether the differences in rotation speed or direction between target and flanker would affect the spatiotemporal properties of crowding (Bex & Dakin, 2005; Greenwood & Parsons, 2020). Similarly, future studies could vary the location of the target and flankers in terms of both their polar angle and eccentricity to replicate well-known location dependencies using the continuous paradigm.

Future work may also implement alternative analysis techniques such as kernel analysis (Bonnen et al., 2015; Grillini et al., 2020) or deconvolution or modeling approaches similar to those used for pupil size changes (Cai, Strauch, Van der Stigchel, & Naber, 2023; Wierda, van Rijn, Taatgen, & Martens, 2012) that could reveal additional details of tracking performance, such as delay and error distributions. Moreover, combining the continuous psychophysics approach with Quest-like approaches (King-Smith, Grigsby, Vingrys, Benes, & Supowit, 1994; Lesmes, Jeon, Lu, & Dosher, 2006; Lesmes, Lu, Baek, & Albright, 2010; Pelli, 1987; Vul, Bergsma, & MacLeod, 2010; Watson, 2017) could potentially optimize the selection of stimulus parameters to present, enhancing the efficiency of data collection.

Real-world environments frequently involve dynamic changes in both spatial and temporal domains, where objects move relative to each other or change over time. Therefore, examining the interplay between spatial and temporal crowding could further generalize the utility of our paradigm, which provides a framework to explore how these factors jointly influence perception. Future studies could utilize this paradigm to investigate the mechanisms underlying this interplay in more detail.

## Conclusions

Our findings show that orientation tracking performance and recovery rates from sudden changes in orientation decrease in the presence of flankers. Moreover, the continuous psychophysics paradigm measures crowding extents comparable to those found in a trial-based paradigm and consistent with Bouma's rule. We conclude that our continuous paradigm offers a method for integrally investigating the spatiotemporal aspects of visual crowding.
